# Current Research Trends and Prospects for Yield and Quality Improvement in Sesame, an Important Oilseed Crop

**DOI:** 10.3389/fpls.2022.863521

**Published:** 2022-05-06

**Authors:** Rashmi Yadav, Sanjay Kalia, Parimalan Rangan, K. Pradheep, Govind Pratap Rao, Vikender Kaur, Renu Pandey, Vandna Rai, Celia Chalam Vasimalla, Sapna Langyan, Sanjula Sharma, Boopathi Thangavel, Virendra Singh Rana, Harinder Vishwakarma, Anshuman Shah, Abhishek Saxena, Ashok Kumar, Kuldeep Singh, Kadambot H. M. Siddique

**Affiliations:** ^1^National Bureau of Plant Genetic Resources, Pusa Campus, New Delhi, India; ^2^Department of Biotechnology, Ministry of Science and Technology, Government of India, New Delhi, India; ^3^National Bureau of Plant Genetic Resources, Thrissur, India; ^4^Indian Agricultural Research Institute, Pusa Campus, New Delhi, India; ^5^National Institute for Plant Biotechnology, Pusa Campus, New Delhi, India; ^6^Department of Plant Breeding and Genetics, Punjab Agricultural University, Punjab, India; ^7^International Crops Research Institute for the Semi-Arid Tropics, Hyderabad, India; ^8^The UWA School of Agriculture and Environment, The UWA Institute of Agriculture, The University of Western Australia (UWA), Perth, WA, Australia

**Keywords:** abiotic stresses, biotic stresses, core collection, genome assembly, germplasm, interspecific hybrids, phyllody, *Sesamum indicum*

## Abstract

Climate change is shifting agricultural production, which could impact the economic and cultural contexts of the oilseed industry, including sesame. Environmental threats (biotic and abiotic stresses) affect sesame production and thus yield (especially oil content). However, few studies have investigated the genetic enhancement, quality improvement, or the underlying mechanisms of stress tolerance in sesame. This study reveals the challenges faced by farmers/researchers growing sesame crops and the potential genetic and genomic resources for addressing the threats, including: (1) developing sesame varieties that tolerate phyllody, root rot disease, and waterlogging; (2) investigating beneficial agro-morphological traits, such as determinate growth, prostrate habit, and delayed response to seed shattering; (3) using wild relatives of sesame for wide hybridization; and (4) advancing existing strategies to maintain sesame production under changing climatic conditions. Future research programs need to add technologies and develop the best research strategies for economic and sustainable development.

## Introduction

Sesame (*Sesamum indicum* L., Pedaliaceae) is the oldest known oilseed crop, domesticated nearly 3,000 years ago, and the first known oil consumed by man. It is known as the “Queen of oilseeds,” and an orphan crop because it receives little research attention. However, the demand for sesame seeds has increased in the last two decades due to high oil quality, protein content, antioxidant content, and wide adaptability in extreme climatic and edaphic environments (Myint et al., 2020). The genus, *Sesamum* comprises 30 “accepted” species and 17 species with “unresolved” status (Kalwij, 2012). The basic chromosome numbers for the family, Pedaliaceae are x = 8 or 13, with some tetraploids and octaploids (Kobayashi, 1991). Among the *Sesamum* species, *S. indicum* (2n = 26) is the only widely cultivated species across tropical regions, particularly for its oil and protein. Among the wild species, *Sesamum angustifolium* and *Sesamum radiatum* are cultivated infrequently in the regions of Africa (Ashri, 1998). Occasionally, sesame behaves as a perennial species (Ashri, 2007). Seeds are an important source of oil (44–57%), proteins (18–25%), carbohydrates (13.5%), and ash (5%); they also have medicinal and nutritional value (Myint et al., 2020). Sesame consumption is increasing globally due to changes in consumer lifestyle and health awareness programs. Sesame seed consumption will reach approximately $7,245 million by the end of 2024 (∼$6,559 million in 2018).^1^ Globally, the total annual sesame consumption as food and oil is approximately 35 and 65%, respectively. India ranks fourth in the world in sesame seed consumption after Tanzania, China, and Myanmar (Myint et al., 2020). Tanzania remains as the largest producer of the sesame seed worldwide (14.6%), followed by Myanmar (12.78%) and India (12.4%).

Globally, sesame is cultivated on 11.7 million hectares (Mha), producing 6.02 million tons (Mt) with an average seed yield of 512 kg ha^–1^. Continent-wise, Asia and Africa are the largest producers of sesame seeds (97%), followed by North America and Europe. European countries demand high imports of sesame seeds from other countries (Islam et al., 2012). India is the largest producer of sesame seeds, followed by Myanmar and China. Myanmar contributes approximately 9.5% of sesame exports globally. Recent (Food and Agriculture Organization Statistical Databases [FAOSTAT], 2018) data showed that China (1,223 kg ha^–1^) has the highest sesame seed yields, followed by Nigeria (729 kg ha^–1^) and Tanzania (720 kg ha^–1^) (Myint et al., 2020).

Global oil production [214 Million Metric Tons (MMT)] from sesame (5.5 MMT) lags behind groundnut, soybean, rapeseed, sunflower, and linseed.^2^ By 2030, sesame oil consumption is estimated to be approximately 100 MMT globally (Troncoso-Ponce et al., 2011). However, the global cultivated area of sesame has recently declined, mainly due to shifts in cultivation to other cash crops and due to the exposure of sesame crops to many biotic and abiotic stresses. Sesame cultivation is currently limited by low yields due to lack of production strategies. Thus, there is an urgent need to investigate the potential of sesame as a genetic resource for improving sesame production. This study reviews the challenges for sesame production and reveals its potential uses and prospects.

## Conservation of Sesame Germplasm Worldwide

Improving sesame yield and quality is possible by exploring and using its genetic diversity. Sesame germplasm resources offer a broad genetic foundation for breeding (Dossa et al., 2017a). However, studies on the genetic diversity of sesame germplasm are not available, with only a few studies available on developing phenotypically desirable variable mutants through induced mutagenesis (Benson et al., 2013). As the primary center of origin, Indian sesame cultivars have broad variability for different agro-morphological traits; nevertheless, there is limited information on the genetic diversity in Indian sesame. Developing a core set of sesame germplasm (generally 10% of an entire collection) is efficient for accessing available genetic diversity (Iqbal et al., 2018). However, a major limiting factor for productivity is the narrow genetic base with single genotypes subjected to mutation and selection to develop cultivars. An alternative might be to use existing variability conserved in gene banks rather than inducing variability in existing cultivars. Such variability in crop germplasm, including wild relatives, could be used in pre-breeding programs to broaden the genetic base of various desirable traits for introgression into existing cultivars.

Earlier studies classified the genus, *Sesamum* based on morphological and microscopic observations. *Sesamum orientale* var. *malabaricum* and *Sesamum mulayanum* belong to the same taxon. A wild progenitor of sesame, *Sesamum indicum* subsp. *malabaricum*, is only found on the Indian subcontinent, evidenced by morphological, cytogenetical, and molecular closeness (Hiremath and Patil, 1999; Kawase, 2000; Nanthakumar et al., 2000). In 2004, the International Plant Genetic Resource Institute (IPGRI) and National Bureau of Plant Genetic Resources (NBPGR) recognized 17 species (23 taxa, including intraspecific) in the genus *Sesamum*, mostly African species (IPGRI and NBPGR, 2004). Later, *Sesamum laciniatum* Willd. was merged with *Sesamum prostratum* Retz., reducing the species rank of *Sesamum malabaricum* to subspecies under *S. indicum* (Bedigian, 2015).

**FIGURE 1:**
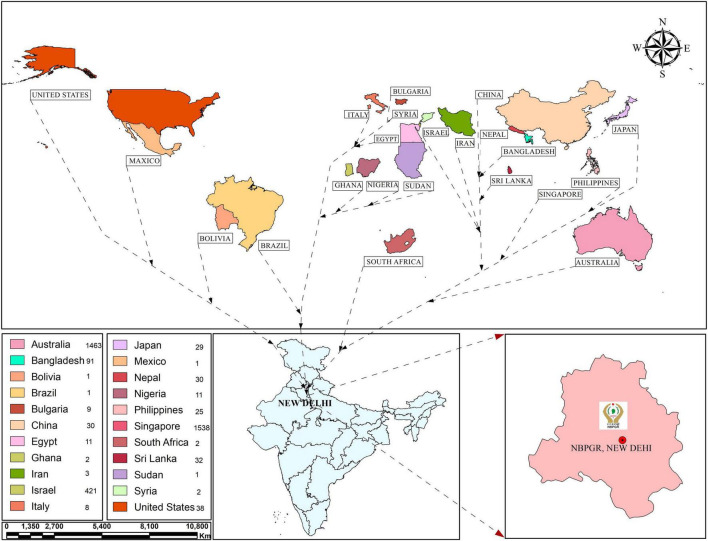
Sesame germplasm exotic collections (ECs) in India from different countries.

Assessing genetic diversity is paramount in crop improvement programs. Studies involving fewer than a few thousand cultivars/landraces/genotypes have been reported, especially for morphological traits. The earlier study investigated 12 agronomic traits in 2,246 sesame accessions from ten agroclimatic zones preserved in the Rural Development Administration (RDA) Genebank in Korea, establishing a core collection of 475 accessions (Kang et al., 2006). To date, 7,698 sesame accessions are stored at RDA (Park et al., 2015). Park et al. (2015) established a core collection of 278 sesame accessions at RDA Genebank from 2,751 accessions collected from 15 countries. The core set was prepared based on 10 quantitative and five qualitative traits. Similarly, Laurentin and Karlovsky (2006) carried out genetic diversity relationship studies in Venezuelan sesame germplasm (32 accessions collected from five countries). The ICAR-NBPGR, New Delhi, India, has 9,625 conserved accessions (as of December 2, 2021) of sesame germplasm in the National Genebank (NGB), including 7,217 indigenous collections (ICs) and 2,408 exotic collections (ECs) (Figure 1). These accessions were procured from various sources, mainly through the International Board of Plant Genetic Resources, Rome, Italy (now Bioversity International) supported program and world collection based in Israel. Bisht et al. (1998) reported diversity in an Indian sesame collection (3,129 accessions), stratifying the accessions into seven diversity groups, developing a core set of 172 accessions. Similarly, other workers on sesame collected 2,168 sesame germplasm from 14 countries and developed a core set of 343 accessions (Mahajan et al., 2007). Wide variation has been reported for quantitative variables, such as yield per plant, capsules per plant, seeds per capsule, height, locule number and arrangement, and the number of primary and secondary branches. Another study revealed substantial variability in *S. indicum* and *S. mulayanum* (now *S. indicum* subsp. *malabaricum*) germplasm (72 accessions) based on agro-morphological and molecular characters (Sarkar and Saha, 2013). The key traits associated with sesame and its closely related wild species are listed in Table 1. Pandey et al. (2015) reported morphological and genetic diversity in 60 sesame accessions collected from different parts of the world (India, Bangladesh, Bulgaria, and United States) based on molecular and phenotypic data. The global genebanks/seed banks procuring sesame accessions are provided in Figure 1, with India, China, and South Korea among the largest sesame germplasm holders. Further, exploration is needed to collect locally available sesame accessions for efficient genetic diversity conservation.

**TABLE 1:** Different sesame species endowed with specific key traits.

Section	Taxon (synonyms)	Chromosome no. (2n)	Distribution and habitat	Key diagnostic characters	Traits of interest
*Sesamopteris* Endl.	*S. alatum* Thonn. (*S. ekambaramii* Naidu)	26	Nigeria, Sudan, Mozambique; introduced weed in Andhra Pradesh, Karnataka, Tamil Nadu; found on sandy soils, in riverbeds, grasslands, and as a weed	Erect, annual, branched, lower leaves palmately divided, upper leaves linear to lanceolate, corolla pink to carmine, nectary sessile, capsule with tapering pointed beak and paper-thin walls, seeds membranous-winged, blackish	Resistant to phyllody, has been transferred to sesame; free from leaf webber and powdery mildew; good plant type, high number of seeds/capsules
*Sesamum*	*S. indicum* L. subsp.*indicum* (*S. orientale* L., *S. trifoliatum* Mill.)	26	Tropics to subtropics	Erect, annual, simple or branched, leaves variable, basal leaves mostly trifoliolate, corolla campanulate, whitish/violet/pink, extrafloral nectaries 1–1.2 mm in diameter. Seeds black/brown/tan/beige/rust-red/mustard-yellow/ivory/white, (mostly) smooth or granular; seed edge sloping; non-dormant seed	
	*S. indicum* L. subsp. *malabaricum* (Burm.) Bedigian (*S. malabaricum* Burm.; *S. mulayanum* N.C. Nair)	26	India (except NE India); from plains, roadsides, railroad tracks, cultivated fields, and hilly forests at elevations to 1,600 m. Often weedy populations occur on roadsides	Erect, annual, branches, many leaves deeply dissected/divided with dentate margin, basal leaves mostly trifoliolate, flowers white to pale purple, with deep purple hue. Lower lip dark purple. Extrafloral nectaries 2–3 mm in diameter, yellow. Strictly bicarpellate capsule. Seeds black/dark brown, surface reticulate and rugose, seed edge acute. Often with markedly purple tinted leaves, stems, petioles and corolla. Dormant seeds	Donor for *cms*; free from leaf webber and powdery mildew; withstands high rainfall; tolerates waterlogging. According to Hiremath and Patil (1999), wild types of sesame might have higher seed oil content and better fatty acid profile than cultivated sesame
*Chamae-sesamum* Benth.	*S. prostratum* Retz. (*S. laciniatum* Willd.)	32	India (Deccan Hills), Africa; prefers sandy tracts, coastal sandy areas, waste grounds	Prostrate, perennial; leaves small, blade irregularly undulate/dentate, leathery; flowers purplish red; capsules leathery, lignified, open with difficulty, seeds without wings, testa pitted	Resistant to phyllody, leaf webber, and powdery mildew; tolerates drought, salinity, and seed shattering
*Aptera* Seidenst.	*S. radiatum* Schum. & Thonn. (*S. occidentale* Heer & Regel)	64	Africa, Madagascar, Upper Guinea, Sri Lanka; Indonesia, naturalized in Kerala and Tamil Nadu	Erect, annual, simple or branched, leaves entire (including basal leaves), sub-opposite, blade ovate to elliptical, extrafloral nectaries purplish, corolla pubescent, pink to purplish, capsule short, with a very short beak at apex, often with two short lateral protuberances, seeds blackish, smooth, margined, testa with radial sculptures	High number of capsules and wide adaptation; drought-tolerant, resistant to powdery mildew, leaf webber, and phyllody

## Significant Advances in Genomics and Genetics

### Genome Sequence Resources

Sesame is a member of the family, Pedaliaceae with 16 genera. The genus, *Sesamum* comprises nearly 37 species distributed across Africa, India, East Indies, and Australia. Cultivated sesame, *S. indicum* L. (syn. *Sesamum orientale* L.), is a diploid species (2n = 2x = 26) with an annual crop duration of 70–150 days. Four *Sesamum* species have been characterized as tetraploids (2n = 4x = 32) [*Sesamum angolense*, *S. angustifolium*, *S. laciniatum*, and *Sesamum prostratum*] and three as octaploid (2n = 8x = 64) [*S. radiatum*, *Sesamum occidentale*, and *Sesamum schinzianum*]. The octoploid species were studied cytologically for their genomic relatedness—*S. radiatum* and *S. occidentale* were similar and homologous, with 32 bivalents and fertile hybrids. Closely related species of *Sesamum* are *Ceratotheca sesamoides* and *C. triloba* (2n = 32) (Hiremath and Patil, 2002). The chromosome number of *Sesamothamnus busseanus* is unknown, while *S. triphyllum* and *S. capense* are 2n = 26. *S. tavakarii* is a new species from Maharashtra, India, with further investigations required in this context. For C-values estimated from the Kew Database, the genome size (1C value) of diploid species (2n = 26) ranged from 950 Mb (*S. indicum*) to 1,651 Mb (*S. alatum*), while tetraploids and octaploids (2n = 32 and 64) ranged from 933 to 1,154 Mb and 1,306 to 1,551 Mb, respectively. The release of sesame whole-genome sequence information triggered the functional analysis of candidate genes. *De novo-*based assembly of the sesame genome (29 sesame accessions from 12 countries) revealed 27,148 genes and high genetic diversity in genes related to oil biosynthesis, resulting in variation in sesame oil content (Wang S. et al., 2014).

**FIGURE 2:**
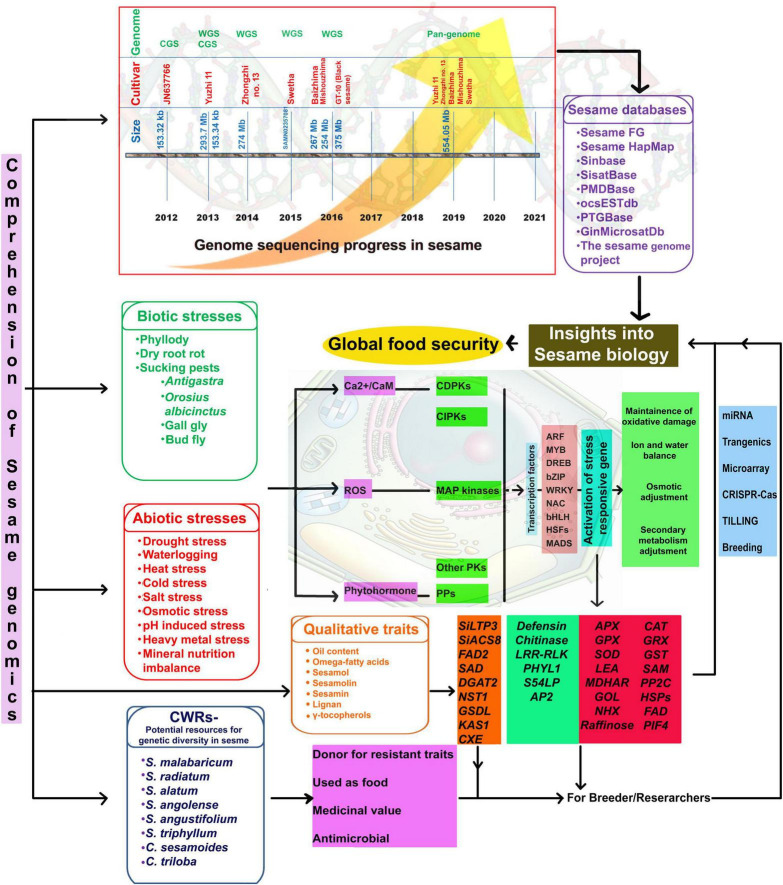
Comprehension of sesame genomics for global food security. CGS, Chloroplast genome sequencing; WGS, Whole-genome sequencing; CWRs, Crop wild relatives; Ca^2+^, Calcium ion; *CaM*, Calmoldulin; ROS, Reactive oxygen species; *CDPKs*, Calcium-dependent protein kinases; *CIPKs*, CBL-interacting protein kinases *PPs*; *MAP kinases*, Mitogen activated protein kinases; *PKs*, Protein kinases; PPs, Protein phosphatases; *ARF*, Auxin response factors; *MYB*, Myeloblastoma; *DREB*, Dehydration responsive elemental binding; *bZIP*-Basic leucine zipper domain; *NAC*, NAM [No apical meristem, ATAF, CUC (Cup-shaped cotyledon)]; *bHLH*, Basic helix loop helix; *Hsfs*, Heat stress specific transcription factors; MADS-M, minichromosome maintenance factor 1 from *Saccharomyces cerevisiae*; A for Agamous (AG) from *Arabidopsis thaliana* (from now on, *Arabidopsis*); D for deficient from *Antirrhinum majus*; and S for serum response factor (SRF); *APX*, Ascorbate peroxidase; *GPX*, Glutathione peroxidase; *SOD*, Super oxide dismutase; *LEA*, Late embryogenesis abundant protein; *MDHAR*, Monodehydro ascorbate reductase; *GOL*, Galactinol synthase; *CAT*, Catalase; *GRX*, Glutathione reductase; GST, Glutathione-S-transferase; *SAMe*, S’adenosyl-l-methionine; *PP2C*, Protein phosphatase 2C; *HSPs*, Heat shock proteins; *FAD*, Fatty acid desaturase; *PIF4*, Phytochrome-interacting transcription factor 4.

However, whole-genome sequencing of black-seeded sesame (variety GT-10) using ABI SOLiD and ion torrent estimated the genome size at 375 Mb (Vaja and Golkiya, 2016). Whole-genome sequence information from a single genotype or cultivar will not indicate genetic diversity. Hence, a pangenome is required to understand the extent of existing genomic variation and associated phenotypic variability (Golicz et al., 2016). Recently, genome assemblies have become available for five sesame varieties, including two landraces (*S. indicum* cv. Baizhima and Mishuozhima) and three modern cultivars (*S. indicum* var. Zhongzhi13, Yuzhi11, and Swetha), providing a valuable resource for comparative genomics and gene discovery (Yu et al., 2019). Pangenome studies revealed nearly 81.52, 81.87, 85.95, and 91.14% of the genome sequence in sesame cultivars, Yuzhi11, Baizhima, Mishuozhima, and Swetha, respectively (Yu et al., 2019). Two draft genomes (both at scaffold level) for Indian sesame are available in the public database—one for cv. Swetha (ASM97556v1; PRJNA219369)^3^ and the other for cv. GT-10 (PRJNA 284826; SAMN 3733426) (Wang S. et al., 2014) (Figure 2). Wei et al. (2017) collected genetic information and combined it with comprehensive phenotypic information in a web-based database (SesameFG) for molecular breeding and genetic improvement in sesame. Other sesame-related databases are listed in Figure 2. Therefore, studies are needed to reveal the full sesame genome sequence; in-depth knowledge of the sesame genome will facilitate the genetic improvement of sesame.

### Molecular Markers in Sesame

Genetic markers play an important role in identifying genetic diversity and important traits present in wild and cultivated species of sesame. This technology has geared plant breeding for genetic gain in many oilseed crop species. The randomly amplified polymorphic DNA (RAPD) technique pioneered in identifying sesame molecular markers (Bhat et al., 1999). RAPD and other markers (AFLP, SSR, and ISSR) had since then used genetic diversity analysis and molecular breeding for association mapping. Genetic diversity studies of sesame have been conducted using RAPD and inter-simple sequence repeats (ISSR) markers (Parsaeian et al., 2011). The amplified fragment length polymorphism (AFLP) technique identified molecular markers associated with the capsule indehiscence trait in a closed capsule mutant (indehiscent) derived from induced mutation (Uzun et al., 2003). High throughput techniques, such as restriction site-associated DNA sequencing (RAD-seq), specific length amplified fragment sequencing (SLAF-seq), RNA-seq, genotyping by sequencing (GBS), are being employed in sesame research for single nucleotidepolymorphisms (SNPs) discovery (Wu et al., 2014; Zhang et al., 2016). Genetic diversity assessments using molecular markers have been undertaken in various sesame-growing countries, including India, Uganda (Sehr et al., 2016), Turkey, China, Cambodia, and Vietnam (Myint et al., 2020). Table 2 lists the different molecular markers used for genetic diversity studies in sesame. Simple sequence repeats (SSRs) are robust molecular markers for genetic diversity analysis, including expressed sequenced tag-SSRs (EST-SSRs), chloroplast SSRs (cp-SSRs) (Sehr et al., 2016), genome sequence SSRs (gSSRs) (Wei et al., 2013; Dossa et al., 2016a), cDNA SSRs (Wang et al., 2012), and ISSRs (Kim et al., 2002). Only approximately 7% of the total available markers (∼100,000) have been validated, with a large proportion available for research. Studies related to SSRs in sesame are in progress. Recently, with the availability of whole-genome sequence (WGS) information, SNP markers have been used to survey sesame populations for marker-assisted breeding (Uncu et al., 2016). Next-generation sequencing data identified SNPs with bi-allelic, co-dominant, and inheritance characteristics. High-density arrays can rapidly genotype several common SNPs across samples for genetic analysis and breeding application, as seen in maize (Unterseer et al., 2014), palm oil (Kwong et al., 2016), pigeon pea (Singh et al., 2020), rice (Singh et al., 2015), soybean (Song et al., 2013), sunflower (Bachlava et al., 2012), and wheat (Wang L. et al., 2014).

**TABLE 2:** Use of molecular markers for genetic diversity assessment in sesame.

Plant material	Marker(s) used	PIC value	Population size	Genetic diversity	References
Morocco sesame	ISSR	0.002–0.350	31	Low	Harfi et al., 2021
Ethiopian sesame	ISSR	0.56–1.00	10	Moderate to high	Mawcha et al., 2020
African sesame (Ghana)	SSR	0.80–0.96	25	High	Adu-Gyamfi et al., 2019
Indian sesame	RAPD	0.510–0.885	47	High	Dar et al., 2017
Chinese sesame	RAPD	0.343–0.897	15	Low to high	Quenum and Yan, 2017
Indian sesame	RAPD	–	9	Low	Anandan et al., 2017
	SSR	0.167–0.867	47	Low to high	Dar et al., 2017
African sesame	SSR	0.51–0.60	48	Moderate	Dossa et al., 2017b
Ugandan sesame landrace	nSSR	0.56	121	Moderate	Sehr et al., 2016
	EST-SSRs	0.26	121	Low	
Indian sesame	RAPD	0.130	44	Low	Kesawat et al., 2015
	ISSR	0.675	44	High	
	SSR	0.404–0.740	44	Moderate to high	
	SSR	0.37–0.74	60	Moderate to high	Pandey et al., 2015
Chinese sesame	SSR	0.40	120	Low	Wu et al., 2014
	In-Del	0.30	120	Low	
Indian sesame	SSRs	0.298–0.912	–	Low to high	Badri et al., 2014
Sudanese sesame	RAPD	–	10	Low	Abdellatef et al., 2008

### Targeting Key Agronomic and Quality Traits Using Functional Genomics

**FIGURE 3:**
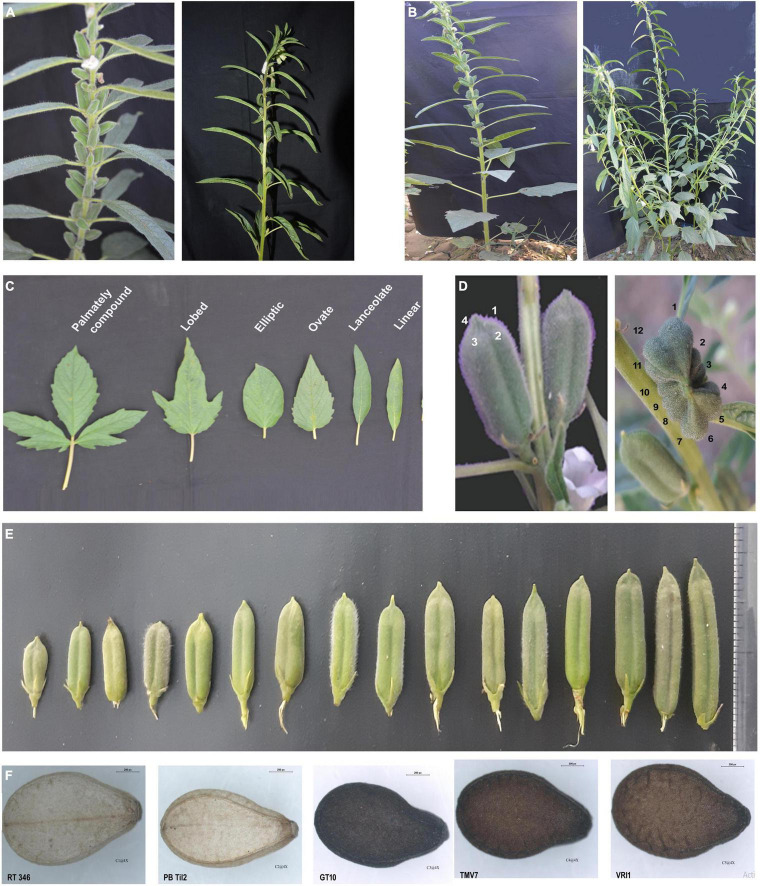
Variability in sesame germplasm for agro-morphological traits. **(A)** Internode stem length, **(B)** branching pattern, **(C)** leaf shape, **(D)** locules per sesame capsule, **(E)** capsule length, and **(F)** seed coat color.

Sesame oil is a sesame seed product with higher unsaturated fatty acids and natural antioxidants, with medicinal, organoleptic, nutritional properties, and superior oxidation stability than other vegetable oils (Takahashi et al., 2016). The oil yield from sesame seeds generally ranges from 35 to 55%, with some cultivars up to 63.2% (Wang S. et al., 2014). Sesame seed oil is rich in omega-6 fatty acids but lacks omega-3 fatty acids, warranting the production of more omega-3 fatty acids, such as alpha-linolenic acids. A comparative study on the fatty acid composition of sesame seed oil from different geographical regions (Morocco, Sudan, Congo, Turkey, and Egypt) revealed variation in palmitic acid (8.5–12.9%), stearic acid (3.0–5.5%), oleic acid (38.9–47.5%), linoleic acid (36.4–46.2%), linolenic acid (0.2–0.4%), saturated fatty acids (14.0–16.3%), and unsaturated fatty acids (83.9–85.1%) (Gharby et al., 2017). Characterizing key genes involved in the metabolic pathway would be important to gain deep insights into sesame oil diversity and other traits. Wei et al. (2015) identified key genes associated with sesame oil composition, pigmentation, and cell wall lignification in 705 accessions. A genome-wide association study (GWAS) identified the novel genes, *SiLTP3* and *SiACS8* related to seed yield traits, such as capsule length and number, but such studies are limited (Wang S. et al., 2014; Zhou et al., 2018). Variation in oil content may arise from seed color (Wei et al., 2015). Similarly, a recent GWAS study on sesame (336 lines in 12 environments) identified 92 potential genes associated with sesame seed coat color (Cui et al., 2021); late-maturing and white-seeded sesame cultivars had higher oil contents than early maturing or black-seeded genotypes, indicating a possible link between oil content and seed coat color. Another study reported increased antioxidant activity in white seeds compared to black seeds (Vishwanath et al., 2012). Capsules at different positions on the same plant can have varying oil contents, with basal capsules containing more oil than apex or branch capsules (Mosidis and Yermanos, 1985). In general, capsules with more carpels would produce more seeds and yield. Germplasm accessions originating from Japan and Far East countries generally exhibit tetracarpellary capsules, while those from other Asian countries mostly have bicarpellary capsules (Ali et al., 2007). Variation in the agro-morphological traits of sesame germplasm is shown in Figure 3. Cultivars with a determinate growth type generally have higher stearic and oleic acid contents and lower linoleic acid than indeterminate types. Environmental factors, especially temperature, strongly influence the fatty acid composition. Determinate sesame genotypes generally have higher oleic acid content and lower linoleic acid content than indeterminate genotypes (Uzun et al., 2002). Thus, non-branching phenotypes might be a good choice for sustained oil content. A combined research effort involving germplasm from different countries could produce sesame genotypes with desired traits.

There is limited information on the biochemical composition of cultivated sesame germplasm. Fifty-four indigenous elite varieties evaluated for oil and fatty acid content contained oleic acid (38–50%) and linoleic acid (18–43%) as major fatty acids but had very low α-linolenic acid (ALA, C18:3, ω-3) (Bhunia et al., 2016). Indian sesame varieties, such as TC-289, PB Til 2, and Var-9 had the highest amount of oleic (50%), linoleic (43%), and α-linolenic acids (1.3%), respectively. Interestingly, Krishna, T-13, and Var-9 contained approximately 1:1 proportions of oleic and linoleic acid. Another study reported a similar percentage range for fatty acid composition using Indian *S. indicum, S. mulayanum*, and *S. radiatum* species (Mondal et al., 2010). NCRIBEN-01M, NCRIBEN-O2M, and NCRIBEN-03L were released in Nigeria for high oil content and white seed color (Olowe and Adeoniregun, 2010). Important genes related to oil biosynthesis in sesame are listed in Table 3. These genes are likely to play a central role in sesame and other oilseed crops and could be used to study the oil biosynthesis pathway, offering new opportunities to investigate novel genes.

**TABLE 3:** Genes for oil-related contents in sesame.

Gene	Function	Trait	References
*CXE17 (Carboxyl esterase 17)*	Encodes lipase and plays an essential role in the oil metabolic pathway	Oil biosynthesis	Wei et al., 2015
*GSDL-like lipase*	Encodes lipase and plays an essential role in the oil metabolic pathway	Oil biosynthesis	
*PPO (Polyphenol oxidase)*	Indirectly involved in oil biosynthesis	Oil biosynthesis	
*NST1*	Belongs to NAC transcription family and indirectly involved in the oil metabolic pathway	Oil biosynthesis	
*KASI (Keto-acyl carrier protein synthase)*	Maintains fatty acid composition through palmitic acid biosynthesis in endoplasmic reticulum	Oil biosynthesis	
*DGAT2 (Diacylglycerol acyl-transferase)*	Maintains fatty acid composition through triacylglycerol biosynthesis in endoplasmic reticulum	Oil biosynthesis	
*FAD2 (Fatty acid desaturase 2)*	Predicted to encode oleic acid desaturase, which converts oleic acid to linoleic acid in endoplasmic reticulum	Fatty acid biosynthesis	
*SAD (Stearoyl-acyl-carrier-protein desaturase)*	Fatty acid biosynthesis by increasing oleic acid content	Fatty acid biosynthesis	
*Tocopheral cyclase*	Converts various phytyl quinol pathway intermediates to their corresponding tocopherols	γ-tocopherol biosynthesis	Pathak et al., 2014
*γ-tocopherol methyltransferase*	Synthesizes vitamin E from γ-tocopherol	α-tocopherol synthesis	
*Δ12 and Δ15 desaturases*	Synthesis of omega-6 and omega-3 fatty acids by adding a double bond at the 12th and 15th carbon position	High linoleate or linoleneate content	
*Sterol 24C methyltransferase*	Converts S-adenosyl-L-methionine to S-adenosyl-L-homocysteine	High β-sitosterol content	

Previous reports identified genetic variation in sesame using agro-morphological traits, such as plant height, primary and secondary branch numbers per plant, internode length, stem height to first branch, day to flowering, capsule number per plant, seed number per capsule, and yield per plant. Several agro-morphologically important traits of sesame are listed in Table 4. Seed color, seed size, and seed shattering are traits associated with domestication syndrome (Bartel and Fink, 1995), defined as the loss of the seed dispersal mechanism in plants, where seeds remain attached to the parent plant for easy harvest. However, mature sesame capsules shatter seeds on their own—a genetically controlled mechanism—limiting crop production. The anatomical basis of capsule shattering has been reported for 32 sesame varieties (Hiremath and Patil, 2002); two important enzymes [cellulase and polygalacturonase (PG)] partly control dehiscence in sesame. Thus, selecting genotypes with low cellulase and PG activity has the potential for breeding genotypes with the non-shattering trait.

**TABLE 4:** Genes related to morphological traits in sesame.

Gene	Function	Trait	References
*ACS (1-amino-cyclopropane-1-carboxylate synthase)*	Auxin-induced gene involved in ethylene biosynthesis	Capsule phenotype and leaf width	Wei et al., 2015
*DOG1 (Delay of germination)*	Regulates seed germination and anthesis	Flowering	
*IAA14 (Indole-3-acetic acid)*	Regulates anthesis	Flowering	
*DFL1 (Dwarf in light 1)*	Negatively regulates cell elongation, lateral root formation, and hypocotyl length	Plant height and oilseed yield	
*GL3 (GLABRA 3)*	Flower lip color development by mediating anthocyanin biosynthetic pathway	Flower lip color	
*ILR1 (IAA* leucine resistant 1)	Codes for aminohydrolase which modulates the activity of auxin in auxin signaling pathway	Plant height and oilseed yield	Bartel and Fink, 1995
*GI (GIGANTAE)*	Regulates the expression of anthesis related genes controlled by photoperiod	Flowering	Fowler et al., 1999
*SiCL1 (Curly leaf 1)*	Encodes a transcription repressor KAN1 (KANADI1) protein which regulates abaxial identity, leaf growth, and meristem formation	Leaf curling and capsule indehiscence	Zhang, 2018

Sesame is considered a small factory of potent molecules. Sesame oil contains important bioactive metabolites, including tocopherols and lignans, that contribute to human health (Mancebo-Campos et al., 2014). Tocopherols prevent the oxidation of sesame oil. γ-tocopherol is a major compound in sesame oil (∼90.5%), while other tocopherols (α, β, δ) account for less than 5% of the total content (Li et al., 2011). Another important sesame metabolite is lignan, formed by the oxidative coupling of p-hydroxyphenylpropane; the major lignans in sesame seeds are sesamin, sesamolin, and sesamol (Shi et al., 2017) (Table 5). Sesamol protects cell membranes from oxidative damage (Chennuru and Saleem, 2013). γ-tocopherol and lignans have cholesterol-lowering, neuroprotective, anticarcinogenic properties, and coronary protective effects (Kim et al., 2016). γ-tocopherol and lignans prevent peroxidation of cell membranes, increasing the rate of fatty acid oxidation in peroxisomes. Sesamin reduces the neurotoxic effect of microglial activation (mediated through lipopolysaccharide), increases the vitality of neuronal cells, and helps mitigate brain injury by lowering the levels of the mediator compound responsible for inflammation (Udomruk et al., 2018). Thus, sesamin could be used to treat serious brain diseases, such as Parkinson’s and Alzheimer’s disease. Other studies have reported the role of sesamin in curing disorders, such as osteoarthritis, ischemic brain stroke, acute hepatic injury, hepatic failure, and diabetic retinopathy, using rats as the model organisms (Wu et al., 2019). By 2035, the estimated number of cancer cases will be approximately 24 million per year, with 14.6 million associated deaths (Stewart et al., 2016). Therefore, sesamin is a potential adjuvant therapeutic agent for developing tumors and could be used to treat/prevent various types of cancer (Majdalawieh et al., 2017). Limited progress has been made using desirable traits through plant breeding due to the lack of appropriate genomic tools. However, breakthroughs in “omics” may advance sesame breeding.

**TABLE 5:** Genes involved in lignin biosynthesis in sesame.

Gene	Function	Trait	References
*Sesamin synthase*	Encoded by gene *CYP81Q1*, involved in primary and secondary metabolic pathways	Sesamin biosynthesis	Chandra et al., 2019
*CYP92B14*	Responsible for sesamin oxygenation to form sesamolin and sesaminol	Sesamolin biosynthesis	Murata et al., 2017

## Strategies for Enhancing Stress Resistance in Sesame

### Enhanced Abiotic Stress Resistance in Sesame

Various abiotic stresses threaten sesame production, including drought, waterlogging, salinity, and high temperature. Stress signals are received by specific receptors present on the plasma membrane, including ion channels, G-protein coupled receptors (GPCRs), and kinases, such as receptor-like kinases and histidine kinases. GPCRs are considered the most prominent signal, activating downstream signaling. Once the receptor is activated upon stress or signal induction, it regulates calcium ion concentration inside the cell and secondary messengers, such as abscisic acid (ABA), reactive oxygen species (ROS), and inositol phosphates. This ultimately activates various transcription factors (TFs), such as MYB/MYC, dehydration responsive element binding protein (DREB), basic leucine zipper domain (bZIP), WRKY by turning on protein kinases, phosphatases, and calcium-dependent protein kinases (CDPKs). The TFs activate myriad novel/unknown and known genes, such as ascorbate peroxidase (APX) (Padaria et al., 2014), heat shock proteins (HSPs) (Vishwakarma et al., 2018; Vishwakarma et al., 2019), and osmolytes (e.g., proline), to save the cell from desiccation. Table 6 describes various genes/TFs involved in the abiotic stress tolerance of sesame. Biochemical changes in stressed plants can be detected by measuring relative water content, total chlorophyll content, malondialdehyde (MDA) content, proline content, hydrogen peroxide (H_2_O_2_) levels, superoxide dismutase (SOD) activity, among others. A few studies have attempted to understand sesame tolerance to different abiotic stresses by systematically screening germplasm and the core collections in Korea (Park et al., 2015), India (Bisht et al., 1998), and China (Zhang et al., 2000). Various states in India have released 89 sesame varieties, with particular phenotypic traits and biotic and abiotic stress tolerance. Madhya Pradesh contributed 11 varieties, followed by Orissa (10), Tamil Nadu (9), Rajasthan, Gujarat, Maharashtra, and Andhra Pradesh (8 each), Kerala (7), Uttar Pradesh, Punjab, West Bengal, and Karnataka (4 each), Haryana (2), and Bihar and Himachal Pradesh (1 each) (Ranganatha et al., 2013). Dossa et al. (2016a) emphasized the need for more phenotypic and molecular analysis of African germplasm for stress-tolerant traits. Several recent studies have used transcriptomics to identify TFs, genes, and miRNAs associated with various abiotic stresses in sesame (Zhang et al., 2020). Major abiotic stresses affecting sesame are discussed below.

**TABLE 6:** Genes for abiotic stress tolerance in sesame.

Gene	Function	Abiotic stress	References
*bZIP*	Multiple biological processes, including anthesis, seed maturation, embryogenesis	Drought, salinity, heat, and waterlogging stress	Wang et al., 2018
*WRKY*	Plant growth and development	Waterlogging stress	Li et al., 2017
*ABC transporter*	ATP-binding cassette transporters	Salinity stress	Rasheed et al., 2018
*Beta-glucosidase*	Hydrolysis of glycosidic bonds	Salt stress	
*LEA gene*	Late embyogenesis abundant protein	Salt stress	
*Glutamate decarboxylase*	Involved in GABA synthesis	Salt stress	
*Galactinol synthase 2*	Biosynthesis of raffinose family oligosaccharides	Salt stress	
*Mtld*	Biosynthesis of mannitol	Salt stress	
*NHX*	Na^+/^H^+^ transporter gene	Salt stress	
*Hsf gene family*	Transcriptional activation	Heat stress	Dossa et al., 2016c
*OLP (Osmotin-like protein)*	Maintains cellular osmolarity by compartmentalizing solutes, altering metabolites, and activating reactive oxygen species (ROS) scavenging system	Oxidative stress	
*AP2-ERF DREB*	Binds to specific DRE/CRT elements	Drought stress	Dossa et al., 2016b
*MYB*	Phytohormone signal transduction	Drought and waterlogging stress	Mmadi et al., 2017
*NAC*	Multiple biological processes, such as anthesis, cell cycle control, morphogenesis	Waterlogging stress	Zhang et al., 2018
*GolS (Galactinol synthase) and Raffinose synthase (RS)*	Biosynthesis of oligosaccharide raffinose; ROS scavenger	Waterlogging stress	You et al., 2018
*NCED (9-cis-epoxycarotenoid dioxygenase)*	Possible key enzyme in ABA biosynthesis	Drought stress	
*Lysine-ketoregulate reductase (LKR), Sacchropine dehydrogenase (SDH)*	Amino acid metabolism (lysine)	Drought stress	
*Aminoadipic semialdehyde dehydrogenase (AASADH)*	Amino acid metabolism (lysine) through the saccharopine pathway	Drought stress	
*Genes coding for ROS-scavenging enzymes: super oxide dismutase (SOD), catalase (CAT), ascorbate peroxidase (APX), glutathione-S-transferase (GST), glutathioredoxins (GRX)*	Removing ROS that cause oxidative damage to cells	Abiotic stresses, particularly drought	
*ABI4 (ABA insensitive 4)*	Biogenesis of ABA and gibberellins	Drought stress	Dossa et al., 2019a
*TTM3 (Triphosphate tunnel metalloenzyme 3)*	Root development	Drought stress	
*NIMIN1 (NIM1-Interacting 1)*	Plant defense response	Drought stress	
*SAM (S-adenosylmethionine synthetase)*	Modulates polyamine (putrescine, spermidine, spermine) levels and ROS homeostasis	Drought stress	
*bHLH (Basic helix loop helix)*	Regulates multiple genes	Osmotic stress	
*PP2C (Protein phosphatase 2c)*	Catalyzes the dephosphorylation of substrate and participates in various signaling pathways in plants	Drought stress	Dossa et al., 2017c

#### Drought Stress

Sesame is a rainfed crop cultivated primarily in arid and semi-arid areas and is thus prone to terminal and intermittent drought. Drought stress (DS) mainly occurs in the arid and semi-arid areas of Africa, America, and Asia. Sesame is typically considered a drought-tolerant crop compared to other oilseed crops (Langham, 2007). However, intense and prolonged drought stress during anthesis has deleterious effects on flowering, capsule and seed formation, seed yield, and ultimately oil quality (Dossa et al., 2017b). Prolonged DS at the seedling stage can increase plant mortality. For sesame, long drought stress at the vegetative stage is the most devastating factor, reducing the average yield. There are few systematic studies on tolerant and susceptible sesame genotypes. In a recent study, integrated transcriptomics and metabolomics were used to study the differential expression pattern of metabolites (e.g., proline, cGMP, adenine, guanine, benzoic acid, and putrescine) in drought-tolerant and drought-susceptible sesame genotypes (Dossa et al., 2017d; You et al., 2019). Several earlier studies revealed the role of micronutrients, such as silica, molybdenum, and boron, in providing drought stress tolerance to plants (Rana et al., 2020). A recent study showed that selenium applied to sesame leaves (50-day-old plants) improved DS tolerance by enhancing proline accumulation (Thuc et al., 2021a). In another report, exogenous application of phytohormones [salicylic acid (SA) and kinetin] on sesame plants induced DS resistance by increasing the endogenous phytohormone levels (Hussein et al., 2015). Therefore, studies related to metabolomics should find key differences between drought-sensitive and drought-tolerant genotypes to establish links between phenotypic (morphological level) and genotypic (DNA level) changes in plants under DS (Llanes et al., 2018). DS can be induced artificially using polyethylene glycol (PEG) solution to study morphological and physiological changes in sesame. Earlier studies revealed the role of PEG (in varying concentrations) in examining DS tolerance in sesame plants (Liang et al., 2021). However, only one report has evaluated sesame genotypes under DS (varying PEG concentration) using mutant plants at the seedling stage, establishing two sesame mutants “ML2-5” and “ML2-10” tolerant of severe DS (Kouighat et al., 2021).

Very few molecular studies have been conducted in sesame under DS. Stress-related TFs are essential for plant survival under DS. Evolutionary analysis of important TFs families, such as heat stress-specific transcription factors (Hsfs) and AP2/ERF, revealed that most of the genes in these families were retained during evolution. The conservation of these families is linked to high drought tolerance of sesame. The use of RNA-seq technologies led to the discovery of 61 candidate genes and 722 known genes involved in the drought response (Dossa et al., 2017c). More studies are being implemented to dissect the DS response pathways in sesame.

#### Waterlogging Stress

Sesame is highly susceptible to waterlogging, a major threat to sesame production in South and East Asia, particularly China and Korea (Wang et al., 2016). Even short periods of waterlogging reduce sesame growth and development, leaf area index, chlorophyll content, plant dry matter accumulation, and capsule number, and increase the number of aborted seeds, thus decreasing the yield (Sarkar et al., 2016). Saha et al. (2010) reported that sesame is more sensitive to waterlogging at the reproductive stage than the vegetative stage, reducing seed yield by approximately 44% (3.6–6.4 g plant^–1^) compared to control conditions (6.5–9.9 g plant^–1^).

Only a few studies have unraveled the genetic basis of the waterlogging stress response in sesame (Zhang et al., 2014). A comparative RNA-seq-based analysis between waterlogging-tolerant and susceptible sesame genotypes revealed 66 potential genes for improving sesame tolerance to waterlogging stress (Wang et al., 2016). Another study suggested different underlying molecular mechanisms due to the contrasting response of sesame genotypes under waterlogging stress, which were correlated further with DNA methylation patterns (Dossa et al., 2019b). Upregulation of genes in the bZIP family (e.g., *SibZIP03*, *SibZIP04*, *SibZIP30, SibZIP44*, and *SibZIP62*) might play an important role in metabolic reprogramming under waterlogging stress (Wang et al., 2018). Earlier studies have explored WRKY TFs to understand the structure and function of sesame *WRKY* genes, concluding that manipulating these genes could enhance waterlogging and DR (Li et al., 2017). Other genes related to waterlogging stress tolerance are listed in Table 6. Cultivar Zhongzhi No. 13 is a waterlogging-tolerant cultivar that could be used to genetically enhance sesame under waterlogging stress (Wang et al., 2016). A more comprehensive study identified 1,379 genes central to waterlogging stress tolerance, including 66 new candidate genes. A total of 13,307 genes were differentially regulated under waterlogging stress in sesame (Wang et al., 2012). SSR markers (ZM428) and QTL (qEZ10CHL07, qEZ10ZCL07, qWH10CHL09, qWH10ZCL09, qEZ09ZCL13, and qWH09CHL15) have been used in marker-assisted selection (MAS) for waterlogging stress tolerance (Wang et al., 2012). Combined transcriptomics and SSR studies will unveil sesame genomics associated with waterlogging stress tolerance.

#### High-Temperature Stress

High-temperature stress is a major constraint in sesame productivity, especially during anthesis and seed filling. Temperatures of 25 ± 2°C encourage rapid sesame seed germination, initial growth, and anthesis, while approximately 32°C is considered heat stress and greater than 40°C is considered severe heat stress for sesame, affecting fertilization and seed set, and ultimately reducing the sesame yield and quality. Late-sown sesame suffers yield losses of up to 40–50% (Rani and Kiranbabu, 2017). Therefore, thermotolerant sesame genotypes that can compensate for the yield penalty are urgently needed. Rani and Kiranbabu (2017) screened 442 sesame genotypes under high-temperature stress during anthesis, capsule formation, and seed set, identifying four genotypes (JCS 2846, JCS 2892, JCS 3102, and JCS 3258) with the potential to tolerate severe HS (>40°C). Such genotypes could be used in breeding programs to develop high-temperature stress-tolerant genotypes. Heat stress increases the length of the vegetative phase of sesame compared to plants grown under normal conditions (Firmansyah et al., 2012). Therefore, a systematic and comprehensive screening of sesame germplasm under suitable environmental conditions and hotspot locations for individual abiotic stress tolerance is needed.

#### Stress Perception and Signal Transduction Under Abiotic Stress

To cope with various biotic and abiotic stresses, plants have evolved mechanisms to bring changes at the physiological and biochemical levels that are translated into internal signals to activate stress-responsive genes through an interconnected network of signaling cascades. Thus, identifying genes and metabolites is necessary to understand abiotic stress response mechanisms in sesame plants. Abiotic and biotic stresses lead to oxidative bursts inside plant cells that accumulate ROS, such as hydroxyl radical (OH), hydroperoxyl radical (HO^2^), superoxide anion (O^2–^), and alkoxy radical (RO) (Gill and Tuteja, 2010). Plants deal with the increased ROS levels with different enzymes, such as ascorbate peroxidase (*APX*), glutathione reductase (*GR*), superoxide dismutase (*SOD*), catalase (*CAT*), monodehydroascorbate reductase (*MDHAR*), glutathione peroxidase (*GPX*), and glutathione S-transferase (*GST*) (Gill and Tuteja, 2010). The main sites of ROS production are chloroplasts in green plants and mitochondria in non-green parts (Singh et al., 2019) (Figure 2). Recent studies have compared two sesame genotypes (TS-5 and TH-6) under abiotic stress (salinity stress) by measuring oxidative markers, concluding that TS-5 tolerates abiotic stress better than TH-6. Increased membrane damage, as evidenced from MDA level (oxidative marker to measure stress), under salinity stress is also reported for other plant species, such as *Medicago truncatula* and *Ailanthus altissima* (Mhadhbi et al., 2013; Filippou et al., 2014). Anee et al. (2019) reported increased H_2_O_2_ and MDA levels in *S. indicum* cv. BARI Til-4 (waterlogging sensitive) with time (2, 4, 6, and 8 days) under waterlogging conditions. Similar results (enhanced oxidative markers) have been reported for species, such as the Antarctic plant (Park and Lee, 2019), *Sorghum bicolor* (Zhang et al., 2019), and *Solanum lycopersicon* (Rasheed et al., 2018), under waterlogging. In barley, the waterlogging-tolerant genotype (TF58) overexpressed *SOD* and *GST* compared to the sensitive genotype (TF57) under waterlogging stress (Luan et al., 2018). In sesame, increased *SOD* activity during waterlogging stress suggests that *SOD* acts as an ROS scavenger and thus plays an important role in maintaining cell membrane and protein stability. Using different biochemical markers, such as *SOD*, *CAT*, and *GPX*, studies have identified drought-tolerant (SI-1025, SI-205, SI2138-2, KM-13) and drought-sensitive (Prachi, SI-1926, ENT-78-301, Kanaka, IS-607-1-84) sesame genotypes (Hota et al., 2019). Traits related to yield enhancement under normal and stress conditions are listed in Table 7.

**TABLE 7:** Traits related to yield enhancement in sesame under normal and stress conditions.

Traits	Gene/genotype/molecules	Condition	References
Drought stress tolerance	Silica, molybdenum, boron	Stress	Rana et al., 2020
Drought stress tolerance	Selenium	Stress	Thuc et al., 2021a
Drought stress tolerance	Salicylic acid and kinetin	Stress	Hussein et al., 2015
Drought stress tolerance	Sesame mutants “ML2-5” and “ML2-10”	Stress	Kouighat et al., 2021
Waterlogging stress tolerance	*SibZIP03*, *SibZIP04*, *SibZIP30, SibZIP44*, and *SibZIP62*	Stress	Wang et al., 2018
Waterlogging, drought stress tolerance	WRKY TFs (Transcription factors)	Stress	Li et al., 2017
Waterlogging stress tolerance	Cultivar Zhongzhi No. 13	Stress	Wang et al., 2016
Heat stress tolerance	JCS 2846, JCS 2892, JCS 3102, and JCS 3258	Stress	Rani and Kiranbabu, 2017
Salinity stress tolerance	TS-5 genotype	Stress	Mhadhbi et al., 2013
Drought stress tolerance	SI-1025, SI-205, SI2138-2, KM-13 genotypes	Stress	Hota et al., 2019
Waterlogging stress tolerance	SSR marker (ZM428)	Stress	Wang et al., 2012
Waterlogging stress tolerance	QTL (qEZ10CHL07, qEZ10ZCL07, qWH10CHL09, qWH10ZCL09, qEZ09ZCL13, qWH09CHL15)	Normal	
Non-shattering	Paloma, SW 16, SW17, Baco, and UCR3	Normal	Myint et al., 2020
Early maturity (60-80 d)	Ye-Kyaw, Pat-Le-War-Hmyaung, Man-Shwe-Wa, She-Ka-lay, Mai-Thi-hla	Normal	
Monostem	Victoria, Aida, Valya, and Nevena	Normal	
3 capsule/1 capsule	Zhongzhi13/Baizhima	Normal	Wei et al., 2015
Oil content	*SiACNA, SiDGAT2, SiFATA, SiFATB* and *SiSAD*	Normal	

#### Involvement of Heat Shock Proteins and Heat Stress Specific Transcription Factors to Overcome Stress

Heat shock proteins (HSPs) or molecular chaperones are highly conserved proteins that protect plant cells under abiotic stress through protein folding, assembly, and translocation (Huttner and Strasser, 2012). There are five categories of HSPs based on molecular weight: HSP100, HSP90, HSP70, HSP60, and small HSPs. HSPs are major players enabling cell proteins to survive under stress conditions by stabilizing protein folding (Khan et al., 2019). In sesame under abiotic stress, tolerant genotypes have higher expression of HSPs than sensitive genotypes. Changes induced by plants under abiotic stress are controlled by gene expression, and TFs are important in regulating gene expression. Among TFs, heat stress specific transcription factors (Hsfs), MYB, WRKY, NAC, and bZIP are involved in complex and overlapping processes under abiotic stress (Gill and Tuteja, 2010). The Hsf gene family has been studied widely in many crop plants, with 30 *Hsf* genes reported in sesame (Dossa et al., 2016c), 25 in Arabidopsis, rice (Guo et al., 2008), maize (Lin et al., 2011), and Chinese pepper (Jin et al., 2014), 26 in tomato (Yang et al., 2016), 27 in potato (Tang et al., 2016), 29 in Chinese white pear (Qiao et al., 2015), and 35 in Chinese cabbage (Song et al., 2014). So, it can be concluded that the number of *Hsf* genes present is not linked to plant genome size. In a previous study, all 30 sesame *Hsf* genes were expressed in different parts of the sesame plant (the leaves, shoots, roots, and the flowers), suggesting a conserved functional role at various stages of development. Sesame *Hsf* genes were classified into three major groups (A, B, and C), with group B genes being most expressive under abiotic stress and thus could be used to improve the abiotic stress tolerance in sesame (Dossa et al., 2016c).

### Enhanced Biotic Stress Resistance in Sesame

Biotic stress is induced by living organisms, such as fungi, viruses, bacteria, insects, and nematodes. Plants respond to biotic stresses with ROS-induced oxidative bursts, cell wall lignification, and chemical compound releases, such as benzothiadiazole (BTH) and β-aminobutyric acid (BABA). Various plant hormones, such as SA, jasmonic acid (JA), ethylene (ET), and ABA, play a central role in biotic stress signaling. Here, we mainly focus on two devastating diseases in sesame, i.e., phyllody and dry root rot caused by phytoplasma and fungi, respectively (Rao et al., 2015). Phyllody associated with phytoplasma is the major limiting factor affecting cultivation, described more than 100 years ago, first recorded in 1908 at Mirpur Khas, Pakistan (Vasudeva and Sahambi, 1955). Most cultivated sesame varieties are susceptible to phyllody disease, causing severe economic losses. Phyllody disease also occurs in related wild species, including *S. alatıun, S. indicatum, S. occidentale*, and *S. radiatum*, resulting in yield losses up to 34–100% (Ramanujam, 1944; Rao et al., 2015). Symptoms associated with phyllody disease in sesame include phyllody (floral parts replaced with leafy structures), flower virescence (abnormal development of green pigmentation), witches’ broom, shoot tip fasciation, vivipary (seeds or embryos begin to develop before being detached from the parent), and cracking of seed capsules. Phyllody disease has also been reported in the Asian and African countries, including Burkina Faso, Ethiopia, and India (Madhupriya et al., 2015; Rao et al., 2015), Iraq, Israel, Mexico, and Myanmar (Win et al., 2010), Nigeria, Oman, Pakistan, Sudan, and Taiwan (Tseng et al., 2014), Tanzania, Thailand, and Turkey (Catal et al., 2013), and Venezuela and Uganda (Singh et al., 2007).

Several diagnostic methods, including Dienes stains and TEM techniques, can detect phytoplasma bodies in phloem sieve tissues; however, PCR-based assays are more reliable for identifying phytoplasmas associated with the disease (Rao et al., 2015). Four phytoplasma ribosomal groups, 16SrI, 16SrII, 16SrVI, and 16SrIX, are associated with phyllody disease worldwide, of which 16SrI and 16SrII are the most widespread affecting sesame crops in India (Madhupriya et al., 2015). Complete phytoplasma genome sequencing has not been undertaken. The disease spreads *via* leafhopper species, with *Orosius orientalis* as the major vector. Recently a new vector, *Hishimonus phycitis*, was identified from India. Moreover, many weed species host sesame phyllody-associated phytoplasmas (Rao et al., 2015). The cure for disease phyllody is not known, so prevention is paramount. Developing cultivars with durable phyllody resistance would be the best management tool and should be an integral component of sesame breeding programs. Since most cultivated germplasm is susceptible to phyllody, exploitation of wild relatives as sources of resistance genes could be viable. Some studies have attempted to confer resistance to phyllody disease in sesame using intra- and interspecific crosses of sesame species, including *S. mulayanum, S. indicum*, and *S. alatum* (Singh et al., 2007).

Epidemiologic studies should also be undertaken to eradicate alternate plant hosts and control insect vectors, preventing the epidemic spread of phyllody in sesame. Identifying true phyllody resistance is important because symptomless infections are often undetectable, and disease-resistant genotypes selected in the field might respond differently under other environmental conditions (Atkinson and Urvin, 2012). Ustun et al. (2017) recently screened 542 sesame genotypes under field and greenhouse conditions to identify phyllody resistance; only two accessions, ACS38 and ACS102, were disease resistant. Phytoplasma infection (phyllody disease) in sesame has been identified and characterized by molecular analysis using the 16S rRNA region of phytoplasma (Junior et al., 2019). Phyllogen (a virulence factor) may induce phyllody in many plant species of angiosperms, gymnosperms, and ferns by inhibiting MTFs (MADS domain transcription factor), a key regulator of flower development (Kitazawa et al., 2017).

Another widespread and destructive disease of sesame is dry root rot, caused by *Macrophomina phaseolina* (Tassi) Goid, a soil-borne Deuteromycetes fungus (Islam et al., 2016). Although *M. phaseolina* is reported to be from North and South America, Asia, Africa, and Europe, this pathogen is more prevalent in subtropical and tropical countries having a semi-arid climate. *Macrophomina* exists in two alternative forms: saprophytic (*Rhizoctonia bataticola*) and pathogenic (*M. phaseolina*). The pathogen causes significant yield losses in sesame, ranging from 50 to 100% (Yan et al., 2021). A few studies have investigated dry root rot resistance or tolerance for commercial cultivar development in sesame (Cummings and Bergstrom, 2013). Bedway and Moharam (2019) screened 86 sesame genotypes and found highly significant variations between genotypes across two seasons for disease-infection percentage and seed yield. A recent transcriptomics comparison between resistant (Zhengzhi 13) and susceptible (Ji 9014) sesame genotypes revealed the disease-resistance mechanism against *Macrophomina phaseolina*, with 52 differentially expressed genes involved in different signaling pathways (Yan et al., 2021).

Another major limiting factor for sesame crop production is sucking pest insects. Larvae of *Antigastra catalaunalis* (sesame leaf rollers) feed on the topmost leaves of sesame, resulting in plant death. If infection occurs at later stages, the larvae penetrate the capsule and feed on the developing seeds. *Orosius albicinctus* (jassid) feeds on soft tissues, such as leaves, causing leaf curling; it is also the major vector for transmitting phyllody disease. Maggots, such as gall fly (*Asphondylia sesami*) and bud fly (*Dasineura sesami*) affect sesame floral buds by forming gall-like structures. Another pathogen affecting sesame growth is *Phytopthora nicotianae* (Kumari et al., 2019). Of the 186 sesame genotypes screened, 12 had high resistance to Phytophthora. Four *P. nicotianae* isolates (KACC48120, KACC48121, No2526, and No2040) were identified by the Korean National Agrobiodiversity (Oh et al., 2018), but there is little information on sesame resistance to Phytopthora blight. Identifying the molecular mechanisms involved in biotic stress tolerance in sesame would strengthen our understanding of the regulatory mechanisms against biotic stresses. In addition, identifying changes in gene expression would help predict sesame tolerance to biotic stresses. Genes related to biotic stress tolerance in sesame are listed in Table 8.

**TABLE 8:** Genes associated with biotic stress tolerance in sesame.

Gene	Function	Biotic stress	References
*AP2* (Apetala 2)	Defense marker gene for JA/ET pathway; upregulated in sesame infected with *Macrophomina phaseolina*	Root rot	Chowdhury et al., 2017
ERF (Ethylene-responsive factor)	Defense marker gene for JA/ET pathway; upregulated in sesame infected with *M. phaseolina*	Dry root rot	
*Def* (Defensin)	Defense marker gene for JA/ET pathway; upregulated in sesame infected with *M. phaseolina*	Dry root rot	
*Chi* (Chitinase)	Marker gene for SA signaling; upregulated in sesame infected with *M. phaseolina*	Dry root rot	
*TLP* (Thaumatin-like protein)	Marker gene for SA signaling; upregulated in sesame infected with *M. phaseolina*	Dry root rot	
*bHLH*	Basic helix-loop-helix	*Macrophomina phaseolina*	Yan et al., 2021
*LRR-RLK*	Leucine-rich repeat receptor-like kinase	*Macrophomina phaseolina*	
RLK (Serine threonine kinase)	Signal reception and key role in signaling pathway during pathogen recognition	Charcoal rot	
*PHYL1*	phytoplasmal effector causing phyllody symptoms 1	Phyllody	Singh and Lakhanpaul, 2020
*S54LP*	SAP54 Like Protein of Sesame Phyllody	Phyllody	

#### Insect and Pest Resistance in Sesame

Host plant resistance is a key component of integrated pest management as it is environmentally safe, sustainable, and easy to adopt. The attacking pests on sesame are classified into pathogens, predators, and parasitoids. *Antigastra catalaunalis* is a pest that causes leaf webbing in sesame (Gebregergis et al., 2016). Intercropping sesame with green gram, black gram, sorghum, pearl millet, cluster bean, and pigeon pea significantly reduced the damage caused by *A. catalaunalis* (Ahirwar et al., 2009). Common cultural practices assist pest management in sesame, including timely crop sowing, field sanitation, deep plowing during summer, destroying alternate host plants, sowing of guard/barrier crops, such as maize, jowar, and bajra. Studies have reported that releasing parasitoids (*Trichoderma* spp., *Bracon brevicornis*, *Bracon heabator*, and *Campoplex* spp.) and predators (*Eocantheconia furcellata*, *Cicindella* spp., red ant, spiders, and ladybird beetle) in sesame reduced the population growth of *A. catalaunalis*. Conserving existing natural parasitoids (Ichneumonidae and Braconidae) and predators (coccinellids, predatory stink bugs, mantids, and spiders) combined with appropriate microbial pesticides (e.g., *Bacillus thuringiensis* var. *kurstaki*) help manage leaf webber in sesame (Sasikumar and Kumar, 2014). Gall fly, *Asphondylia sesami* Feltis, is another significant crop pest, reducing sesame seed yield by up to 100% in susceptible genotypes (Mehalingam, 2012). The variety, N-32 released in India is tolerant to gall fly. Leafhopper, *Orosius albicinctus*, transmits phyllody disease caused by phytoplasma, infecting sesame crops under field conditions by 0–65.12% (Seemuller and Harries, 2009). The long-term solution for leafhopper control is the development of resistant genotypes.

### Role of Plant Growth Regulators in Combating Stresses

Phytohormones, including SA, JA, ET, and gibberellic acid (GA_3_), play a crucial role in regulating the immune responses of plants (Shah et al., 2021). Studies have shown that SA has a prominent role in limiting oxidative damage caused by ROS and stomatal conductance in sunflower and barley (Habibi, 2012). Treatment with phytohormones or PGRs improves plant growth and productivity. Exogenous application of PGRs, such as SA and kinetin, to sesame improved plant growth and other physiological activities (Llanes et al., 2018). GA_3_ improves the plant growth and yield in many crop plants, including sesame (Vekaria et al., 2017). Foliar application of GA_3_ at 30 ± 5 days after sowing increased seed number per capsule, plant yield, and percentage of oil content. Similarly, spraying GA_3_ on sesame plants increased the branch number and plant height (Thuc et al., 2021b). Similar effects of GA_3_ were observed in *Vicia faba* L. and other leafy vegetables (Ibrahim et al., 2007; Miceli et al., 2019). The growth regulator, Cycocel improved sesame growth better than Paclobutrazol. Paclobutrazol interferes with GA_3_ synthesis, with high concentrations retarding sesame growth (Sarkar et al., 2006), as reported for tomato (Pal et al., 2016). A high concentration of Paclobutrazol (300 mg L^–1^) decreased seed numbers in maize and canola (Kamran et al., 2018), but the same concentration applied to sesame improved dry matter accumulation and seed production and reduced seed shattering (Mehmood et al., 2021). Therefore, studies are needed to optimize the concentration of PGRs for increased sesame seed production under stress.

## Role of Micro Rna in Sesame Yield and Oil Biosynthesis

Micro RNAs (miRNAs) are small (20–35 nucleotides long) non-coding RNAs that play a key role in various biological processes (anthesis, nutrient metabolism, light fluctuation, and biotic and abiotic stresses) by degrading targeted mRNAs (cleavage or translational repression) (Table 9). Usually, miRNAs are considered core components of the complex network-regulating gene expression. Studies have identified miRNAs in many plant species, such as Arabidopsis, wheat, cotton, eggplant, maize, radish, rice, sugarcane, and sesame. Before 2018, there were no reports of miRNAs in sesame (Joshi and Mandavia, 2018). Earlier studies identified a few novel miRNAs in sesame, such as mir447 and mir8140 (Marakali, 2018). However, deep sequencing has opened the door to identifying large numbers of miRNAs. Recent studies identified 302 miRNAs (including 19 novel and 283 known miRNAs) in sesame (Zhang et al., 2020). The miRNA studies combined with degradome and transcriptomics are unique, modern molecular platform for in-depth studies, which have been recently used to identify 65 novel and 220 previously known miRNAs that regulate oil biosynthesis during sesame germination (Zhang et al., 2021). Identifying novel miRNAs would offer insight into the biological functioning of miRNAs and their evolutionary relationship. Sesame miRNAs require further attention with relation to targeted genes involved in signaling and developmental pathways, especially leaf development, transmembrane transport, and biotic and abiotic stress responses.

**TABLE 9:** List of microRNA (miRNAs) observed in sesame and their predicted target.

miRNA	Target	Predicted target description	References
miR5368		Targets HSP21 (21 kDa Heat Shock Protein) involved in counteracting osmotic stress and cell protection	Zhang et al., 2020
miR156/157		Targets *SPLs* (Squamosa Promoter Binding Protein Like) involved in brassinosteroid and auxin signaling	
miR160		Targets *ARFs* (auxin responsive factors) involved in brassinosteroid and auxin signaling affect root and leaf development	
miR-n32		Targets *PLD1* (Phospholipase D1) that interact with G-protein and protein phosphatase to mediate ABA response	
miR-144		Targets *GOT1* that mediate protein translocation at the thylakoid	
miR-408		Targets *TGD4* (*Trigalactosyl diacylglycerol 4*) involved in the ER-chloroplast lipid trafficking process	
miR-396		Targets *GRFs* (growth-regulating factors) that enhance osmoregulation and decrease the level of reactive oxygen species	
miR-395		Targets *APS1* (ATP sulfurylase 1) involved in decreasing cell energy wastage	
miR-166		Targets *ATHBs* (Homeobox leucine zipper protein) that regulate SAM (shoot apical meristem) development	
Sin-miR396a-5p	XM_011079770.2	*Sesamum indicum* growth-regulating factor 1 (LOC105161914), mRNA	Joshi and Mandavia, 2018
Sin-miR5658	XM_011099043.2	*Sesamum indicum* BAG family molecular chaperone regulator 1-like (LOC105176294), mRNA	
Sin-miR5658	XM_011080551.2	*Sesamum indicum* bZIP transcription factor TGA10 (LOC105162512), mRNA	
Sin-miR8030-3p	XM_011096846.2	*Sesamum indicum* probable WRKY transcription factor 70 (LOC105174673), mRNA	
	XM_011094724.2	*Sesamum indicum* transcription factor bHLH13-like (LOC105173075), transcript variant X1, mRNA	
Sin-miR5658		NBS-LRR (Nucleotide-binding site leucine-rich repeat)	
Sin-miR8140	XM_011087380.2	*Sesamum indicum* protein LONGIFOLIA *2*	
	XM_011091156.2	*Sesamum indicum* probable protein phosphatase 2C 4	
	XM_011075157.2	VAN3-binding protein (LOC105158406)	
	XM_020693528.1	RNA polymerase sigma factor sigE, chloroplastic/mitochondrial (LOC105160679)	
	XM_020694796.1	*Sesamum indicum* transcription factorSRM1 (Salt-Related MYB-like transcription factor) like	

## Transgenic Sesame: A Brighter and Sustainable Future of the Crop

Production of transgenic sesame by integrating foreign genes was first reported by Taskin et al. (1999). Still, limited studies have been undertaken on developing the transgenic sesame. The genetic transformation (cotyledon explants) method used the intron interrupted *Gus* gene, with *nptII* as a selectable marker, with a transformation frequency of 1.01% (Yadav et al., 2010). In another study, sesame (6-day-old cotyledons) was transformed using the sonication-assisted *Agrobacterium tumefaciens*-mediated method (Debnath et al., 2014). Several authors have produced transgenic sesame with useful traits, such as increased biosynthesis of phenylpropane, improved abiotic stress tolerance, and increased oil content (Chowdhury et al., 2017; Bhunia et al., 2016) (Table 10). In most transformation protocols, *Agrobacterium tumefaciens* has been used to insert foreign DNA in sesame, with a few studies using *Agrobacterium rhizogenes* for hairy roots transformation (Jin et al., 2005). Increasing the expression level of key genes involved in oil biosynthesis is needed to increase sesame oil content. For example, manipulating genes, such as *oleate Δ12-desaturase* gene, *FAD1*, *FAD2, DGAT1*, and *PDAT1* would enhance oil content in transgenic sesame. The genetic transformation of sesame would open new avenues for sesame crop improvement, especially increased oil content.

**TABLE 10:** List of transgenic sesame developed for different traits.

Gene name	CDS size (bp)	Gene isolated from	Gene function	Trait observed in transgenics developed	References
*PDAT1* and *FAD3*		*Sesamum indicum*	Triacylglycerol biosynthesis	Increased triacylglycerol biosynthesis	Muthulakshmi et al., 2021
*OLP* (*Osmotin-like protein*)	744	*Solanum nigrum*	Maintains cell osmolarity by solute division and structural or metabolic alterations	Improved drought, salinity, and oxidative stress	Chowdhury et al., 2017
*fad3C* (*Fatty acid desaturase*)	1,140	Soybean	Encodes enzyme which introduces the third double bond into linoleic acid precursors to produce ALA (α-linoleic acid)	Improved oil content in seed	Bhunia et al., 2016
*Phytase*		*Aspergillus*	Hydrolysis of phytic acid	Production of phytase protein	Jin et al., 2005
*Calmodulin-4*	450	*Daucus carota*	Activates Ca^2+^ cascade and maintains cells in excitatory state; biosynthetic activity of secondary metabolites	Enhances biosynthetic activities of phenylpropane derivatives	Mitsuma et al., 2004
**Standardization of transformation protocol in sesame**					
*β-glucuronidase*	1,800	*E. coli*	Converts colorless substrate to colored form	Reporter gene	Chowdhury et al., 2017
*Bar (Bialaphos resistance)* gene	552	*Streptomyces hygroscopicus*	Codes for phosphinothricin acetyltransferase, a useful selectable marker	Provides resistance against herbicide “Basta”	Bhattacharyya et al., 2015
*NptII* (Neomycin phosphotransferase) and *Gus (β-glucuronidase)*	795, 1,800	*E. coli*	Provides resistance against antibiotic kanamycin; converts colorless substrate to colored form	Selection marker for plants, reporter gene	Al-Shafeay et al., 2011
*β-glucuronidase*	1,800	*E. coli*	Converts colorless substrate to colored form	Reporter gene for transformation	Yadav et al., 2010
*Gus (β-glucuronidase)*	1,800	*E. coli*	Converts colorless substrate to colored form	Reporter gene for transformation	Taskin et al., 1999

## Sesamum Close Wild Relatives: Value Addition to Food and Medicine

Closely related genera of *Sesamum* include *Ceratotheca sesamoides* (2n = 32) or false sesame, indigenous to Africa. Leaves and flowers are consumed as vegetables, sauces, and soup preparation (Morakinyo et al., 2020). The slimy secretion from leaves (due to glandular trichomes) is used to treat eye conjunctivitis (Bedigian, 2015). Leaves of *Ceratotheca triloba* (Bernh.) Hook. are often used as a vegetable; the plant has medicinal value in Zimbabwe and South Africa. Young shoots and leaves of *S. alatum* are consumed as cooked vegetable in Africa and Sudan.^4^ Roots and leaves are used to treat insect (scorpion, wasps) bites. Root bark is used to treat stomach disorders in the northwestern Burkina Faso. There are no reports of *S. angolense* leaf consumption, but root extracts are used to treat coughs in Tanzania, and the leaves are used to treat smallpox, scabies, and vitiligo. All plant parts (leaves, stems, and flowers) of *S. angustifolium* are consumed in parts of Africa and Kenya. Leaves of *S. radiatum* are used to prepare thick soup, which is good for children’s teeth and bones, in the Ivory Coast, Benin, and Nigeria (Auwalu and Babatunde, 2007). Leaf extract is used to treat swellings, boils, insect bites, skin infections, and leprosy and facilitate childbirth in Nigeria and Ghana.^5^ People living in Darfur (western Sudan region) use the dried leaves of *C. sesamoides*, *S. angustifolium*, and *S. radiatum* in the preparation of meat and fish. Many mucilaginous polysaccharides from Pedaliaceae leaves are used as food and medicine in many countries (Bedigian, 2010), especially in Africa. *Sesamothamnus busseanus* leaves are used as a vegetable in Tanzania. *S. triphyllum* is used to treat epilepsy in South Africa. Leaves of Pedaliaceae members, especially *C. sesamoides* and *Sesamum* spp. are a rich source of carbohydrates and minerals (Ruffo et al., 2002). In India, people generally use sesame oil to help with anxiety and sleep disorders and as an antimicrobial mouthwash (Anilakumar et al., 2014). Roasted sesame seeds are consumed directly. Comprehensive studies are needed to save these beneficial species.

## Proposed Strategies and Vision for Enhancing Sesame Production

In recent years, “omics” tools have revealed large numbers of functional genes associated with key agronomic traits. However, functional validation of these genes in model plant systems using genetic engineering approaches is lacking. Transgenic approaches offer a quick, easy, and cost-effective transformation of genes in sesame. Chowdhury et al. (2017) were the first to genetically transform sesame. Using a transgenic approach, potential genes related to yield enhancement, oil biosynthesis, and stress (biotic and abiotic) tolerance could be used to transform elite sesame cultivars for a deeper understanding of the molecular mechanisms involved. In general, transgenic sesame is not well accepted by the public. Biotechnology methods, such as genome editing/CRISPR-Cas and target-induced local lesions in genomes (TILLING) are needed to validate the available genes related to key agronomic traits. Wild relatives of sesame are a reservoir of candidate genes; therefore, there is an urgent need to characterize wild sesame germplasm, especially African species. Further, identifying genes related to the indehiscence trait and indeterminate growth habit—essential traits affecting the mechanization of sesame harvesting—should be prioritized. No studies have investigated the genes involved in the root system architecture (RSA) of sesame; there is an urgent need to dissect the RSA of sesame as it plays a vital role in adapting sesame to different climatic conditions. Various biotic and abiotic stresses and socio-economic and technological factors increase the gap between farmer yields and actual potential yields. Awareness programs are needed to teach farmers agricultural practices, such as proper sowing, plant spacings, intercropping practices, and reducing pests and diseases. Developing sesame varieties with traits, such as indehiscent capsules (non-shattering), photo-insensitivity, determinate growth habit, and monoculm stems (stems with no branching) for easy harvest would help enhance sesame productivity. Bulgaria has developed four sesame varieties (Victoria, Aida, Valya, and Nevena) for mechanized harvest, the United States has released several non-shattering varieties (Paloma, SW 16, SW17, Baco, and UCR3), and Myanmar has released early maturing (60–80 days) varieties (Ye-Kyaw, Pat-Le-War-Hmyaung, Man-Shwe-Wa, She-Ka-lay, and Mai-Thi-hla) (Myint et al., 2020). The useful bioactive compounds (sesamin, sesamol, and tocopherols) in sesame seeds add value to this crop. Therefore, a deep understanding of the genetic basis of such traits should be a priority for developing nutritionally superior sesame genotypes. Sesame is known to confer stress tolerance, but little information is available on this crop despite being a food security source for humans. Most studies related to biotic/abiotic stress tolerance in sesame have been undertaken in controlled conditions. Field studies would offer a more comprehensive understanding of stress responses, elaborating on the inter-connecting mechanisms involved in stress tolerance. Therefore, sesame crop mutants need to be generated using transgenic or breeding approaches to understand stress physiology in sesame. Limited micro RNA-related studies have targeted specific genes in sesame. Biodiesel is being produced from oilseed crops (such as castor, mustard, soybean); perhaps sesame should be investigated in this regard. Genetic transformation, gene stacking, and genome editing techniques could be used to reveal desirable sesame traits. In addition, hormonal crosstalk should focus on understanding the signaling behavior of SA and ET under waterlogging stress. Moreover, studies related to ribosomal proteins, such as the 30S subunit, should be undertaken to investigate translation-dependent stress-related protein synthesis in sesame, which may further improve sesame seed quality and the sustainability of sesame crops under harsh environmental conditions. Therefore, evaluating the available sesame germplasm for the aforementioned traits is a must for sustainable development. Wild relatives of sesame (*S. alatum, S. radiatum*, and *Ceratotheca* sp.) are resistant to various biotic and abiotic stresses; therefore, the utilization and conservation of such germplasm are urgently required.

**Box 1. Future directions for enhanced yield and quality in sesame.**

1. Genes related to indehiscence trait, monoculm stem, and determinate growth should be identified to improve the mechanization of sesame harvesting.
2. Root system architecture (RSA) of sesame should be investigated for adapting sesame to different abiotic stresses.
3. Farmers should be educated on sesame pests and diseases through awareness programs.
4. More sesame mutants are needed to gain deeper insights on sesame plant physiology.
5. Genetic transformation (transgenic sesame), gene stacking, and genome editing techniques should be used to reveal desirable sesame traits.
6. Exploitation of wild relatives of sesame to introgress resistant genes in cultivated species to produce climate-resilient sesame.

## Conclusion and Future Prospects

Genes related to sesame resistance present a new exemplar in explicating gene interactions with diseases. Wild relatives of sesame are much more disease-resistant than sesame cultivars. Phyllody is a devastating disease in sesame, but the large genome size and polyploidy level in wild relatives make studies on sesame phyllody challenging. In contrast, the small diploid genome, higher oil content, and fewer linked genes make sesame (*S. indicum*) a potential model plant for studying disease resistance and oil biosynthesis. Developing sesame varieties with waterlogging tolerance and high water-use efficiency will benefit sesame production. The development of sesame hybrids carrying potential traits would reveal greater genetic variation. Therefore, hybrid development programs should be intensified to maximize the sesame yield. There have been few transgenic studies in sesame, with no standardized transformation protocol. However, the use of transgenic technology is limited in sesame; there is an urgent need to establish more precise protocols for the efficient transformation of sesame. Current approaches to raise transgenic sesame are not well accepted by the public. The large sesame germplasm available in different gene/seed banks needs to be explored for traits/genes which would improve seed quality and increase sustainability under harsh environmental conditions. Research on sesame is needed to improve yields, plant architecture, disease and pest resistance, and indehiscent capsules (non-shattering trait). Many wild sesame species are endowed with specific traits, such as resistance to biotic/abiotic stresses and other important agronomic traits, which breeders should use to develop sesame cultivars. A well-defined program should be discussed at the national and international levels for distributing sesame seeds developed from such highly concerted efforts.

## Author Contributions

SK and KSin conceptualized the study. RY, PR, KP, GR, VK, RP, CV, SL, SS, BT, VRan, HV, ASh, and ASa wrote the original draft. AK and KSid contributed to writing, reviewing, and editing the manuscript. KSin and KSid contributed to supervision. RY, AK, and KSin contributed to project administration. RY, SK, and KSin contributed to funding acquisition. All authors contributed equally in their respective expertise and agreed to the published version of the manuscript.

## Conflict of Interest

The authors declare that the research was conducted in the absence of any commercial or financial relationships that could be construed as a potential conflict of interest.

## Publisher’s Note

All claims expressed in this article are solely those of the authors and do not necessarily represent those of their affiliated organizations, or those of the publisher, the editors and the reviewers. Any product that may be evaluated in this article, or claim that may be made by its manufacturer, is not guaranteed or endorsed by the publisher.

## Footnotes

1
https://www.mordorintelligence.com/industry-reports/sesame-seeds-market

2
https://www.statista.com/statistics/263978/global-vegetable-oil-production-since-2000-2001/

3
https://www.ncbi.nlm.nih.gov/biosample/SAMN02357081

4 https://www.cabdirect.org/cabdirect/abstract/20177200497 (accessed on December 1, 2020).

5
https://www.cabdirect.org/cabdirect/
